# Supplementary material for: The interactome of intact mitochondria by cross-linking mass spectrometry provides evidence for coexisting respiratory supercomplexes
Source: Mol Cell Proteomics. 2017 Dec 8;17(2):216–32. doi: 10.1074/mcp.RA117.000470 (PMC5795388; doi:10.1074/mcp.RA117.000470)
Supplement: Supplemental Data [file supp_RA117.000470_133922_0_supp_23978_fzffwf.zip › spectra_annotation/mito_DR_spectra_annotation/479-1-1-1-7-1.pdf]

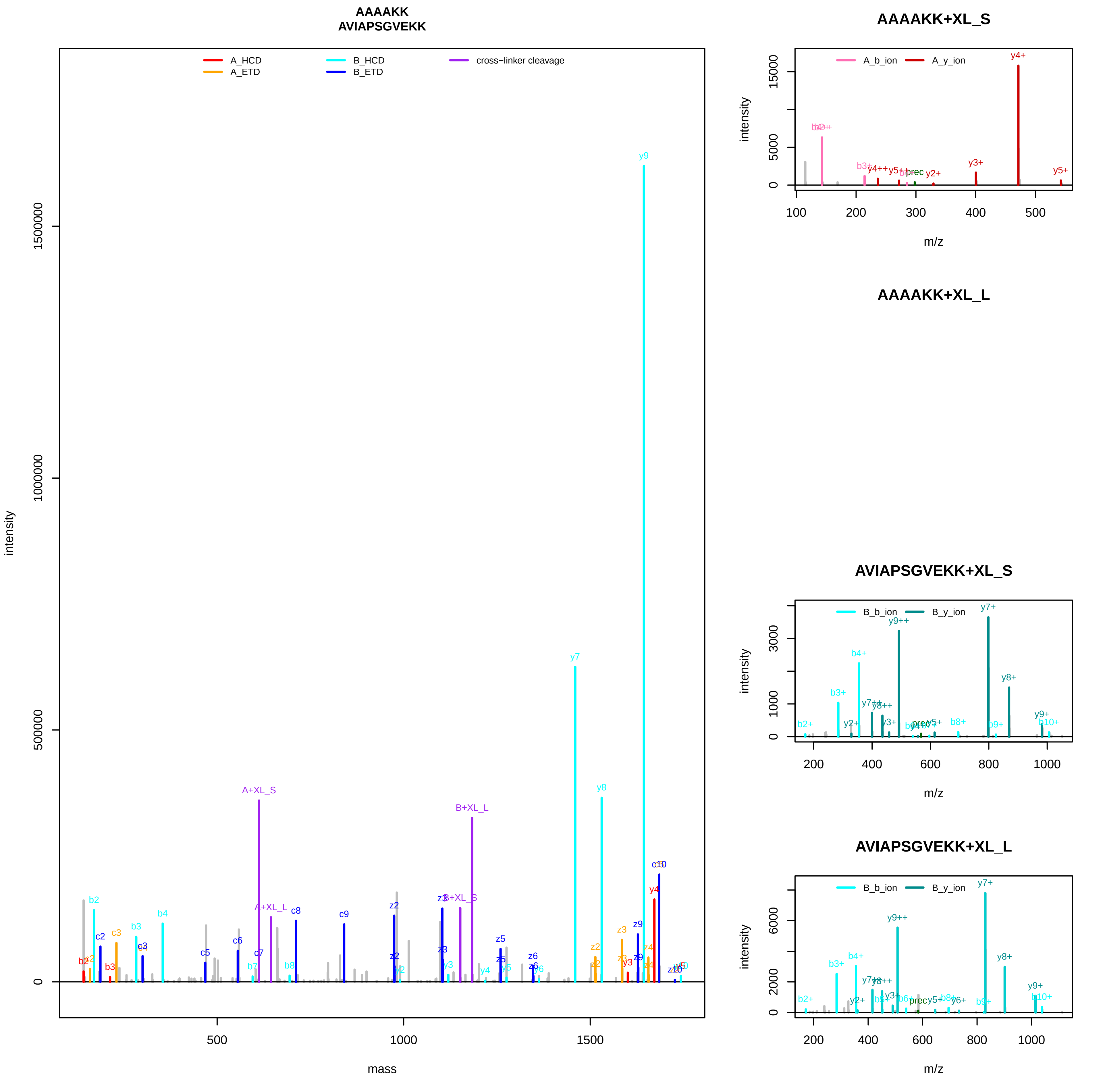
AAAAKK
AVIAPSGVEKK
A_HCD
A_ETD
B_HCD
B_ETD
cross−linker cleavage
intensity
mass
0
500000
1000000
1500000
500
1000
1500
y9
y7
y8
A+XL_S
A+XL_L
B+XL_L
B+XL_S
c10
y4
z9
z3
c9
c8
c7
c6
c5
c3
c2
b2
b3
b4
c3
z2
z5
z6
AAAAKK+XL_S
A_b_ion
A_y_ion
y4+
y3+
y5+
prec
m/z
0
5000
15000
100
200
300
400
500
AAAAKK+XL_L
AVIAPSGVEKK+XL_S
B_b_ion
B_y_ion
y7+
y9++
b4+
y8+
b3+
0
1000
3000
200
400
600
800
1000
AVIAPSGVEKK+XL_L
y7+
y9++
y8+
b4+
b3+
y9+
0
2000
6000
200
400
600
800
1000
